# NLRP3 is crucial for macrophage metabolic reprogramming during *Vibrio vulnificus* infection

**DOI:** 10.1128/spectrum.00230-25

**Published:** 2025-11-05

**Authors:** Ye-Lin Jiang, Xian-Hui Huang, Wen-Hui Zhu, Si-Qi Wei, Jing-Jing Li, Hao-Nan Lin, Huang-Kai Bian, Chen-Lin Wu, Xin-Jun Miao, Yong-Liang Lou, Lu Tang, Dan-Li Xie

**Affiliations:** 1 The School of Laboratory Medicine & Life Science, Wenzhou Medical University 26453, Wenzhou, Zhejiang, China; 2 Key Laboratory of Laboratory Medicine, Ministry of Education of China, Wenzhou, Zhejiang, China; 3 Wenzhou Key Laboratory of Sanitary Microbiology, Wenzhou, Zhejiang, China; 4 Department of Laboratory Medicine, The People’s Hospital of Yuhuan, Taizhou, Zhejiang, China; 5 Department of Emergency, Wenzhou Central Hospital 223520, Wenzhou, Zhejiang, China; 6 Department of Urology, Chinese PLA General Hospital 104607, Beijing, China; University of Guelph College of Biological Science, Guelph, Ontario, Canada

**Keywords:** *Vibrio vulnificus*, NLRP3, glycolysis, metabolism, single-cell sequencing, macrophage

## Abstract

*Vibrio vulnificus* is notorious for inducing rapidly progressive infections with high lethality and significant morbidity. Macrophages, being essential components of the innate immune system, play a vital role in combating infections, and their activation and effector functions are closely intertwined with metabolic reprogramming processes. However, the mechanisms governing macrophage glycolytic metabolism in response to *V. vulnificus* infection remain poorly elucidated. Based on our current data, we propose that NOD-like receptor 3 (NLRP3) could potentially contribute to the regulation of glycolytic metabolism in macrophages infected with *V. vulnificus*, though further validation is needed to confirm this relationship in both *in vivo* and *in vitro*. Upon *V. vulnificus* infection, NLRP3 was shown to augment glucose uptake, upregulate the aerobic glycolytic pathway, facilitate lactate release, and enhance reactive oxygen species (ROS) production. Notably, these metabolic alterations were abolished in NLRP3 knockout (KO) macrophages, as observed in both NLRP3-deficient macrophage cell lines and primary cells. We also found that the abundances of fructose 1,6-bisphosphate and 3-phosphoglyceric acid in glycolytic metabolism were decreased in *V. vulnificus*-infected NLRP3 KO macrophages by non-targeted metabolic flux analysis, which might be due to the reduction of PFKL that converts fructose 6-phosphate to fructose 1,6-bisphosphate in *V. vulnificus*-infected NLRP3 KO macrophage. These findings suggest that NLRP3 may promote inflammation in macrophages during *V. vulnificus* infection by driving glycolysis and increasing ROS production. The absence of these metabolic changes in NLRP3 KO macrophages underlines the crucial role of NLRP3 in modulating the immunometabolic response to *V. vulnificus* infection.

**IMPORTANCE:**

The results of this study demonstrate that NOD-like receptor 3 (NLRP3) is critical for metabolic reprogramming in macrophages during *Vibrio vulnificus* infection. NLRP3 enhances glucose uptake, upregulates glycolysis, reprograms metabolic flux, and promotes reactive oxygen species production. These findings are significant, as they reveal a previously unrecognized role of NLRP3 in regulating immune function in *V. vulnificus*-infected macrophages. This study identifies NLRP3 as a central mediator linking immune cell metabolism to defense against infections, providing novel insights into how innate immunity controls pathogenic bacteria and suggesting potential strategies for improving treatment or prevention of severe infections. However, further research is required to fully elucidate its impact on macrophage glycolysis in *V. vulnificus*-induced sepsis.

## INTRODUCTION

*Vibrio vulnificus*, a halophilic and gram-negative marine bacterium, exhibits a wide distribution in warm, brackish waters globally, with a particular prevalence at river-sea interfaces (1). The infections induced by *V. vulnificus* are distinguished by their abrupt onset, expeditious progression, and substantial morbidity. Clinically, *V. vulnificus* infections predominantly present in three modalities: primary sepsis (accounting for 43.1%), wound infections (45.9%), and gastroenteritis (5%) (2). In the event of primary sepsis, a significant majority of patients experience a precipitous decline in health status, culminating in septic shock and multi-organ failure within a span of 48 h, and the case fatality rate surpasses 50% (3). Notwithstanding the elevated virulence and rapid advancement of *V. vulnificus* infections, the molecular mechanisms underpinning the host defense response remain inadequately elucidated.

Glucose metabolism represents a fundamental metabolic pathway that encompasses a series of enzymatic reactions crucial for maintaining cellular energy homeostasis. This pathway is composed of multiple enzymes that collectively facilitate the conversion of glucose into metabolites and adenosine triphosphate (ATP), thereby supplying the energy required for diverse cellular functions. Through glycolysis, glucose is transported into the cytoplasm, where it is metabolized into pyruvate. Subsequently, pyruvate enters the tricarboxylic acid (TCA) cycle, and the resulting products are predominantly utilized in oxidative phosphorylation (OXPHOS) to generate a substantial amount of ATP (4). Beyond its role in energy production, metabolism plays a pivotal role in regulating the functions of immune cells. Glycolytic intermediates not only serve as an energy source but also play a crucial role in enabling immune cells to adapt their phenotypes in response to external stimuli (5, 6). When resting immune cells are activated, they tend to undergo a metabolic shift from oxidative phosphorylation to aerobic glycolysis, a phenomenon known as the Warburg effect (7). Although glycolysis is less efficient in generating ATP compared to the TCA cycle or OXPHOS, it is an essential metabolic route for immune cell activation (8). Studies have demonstrated that leukocytes in an inflammatory environment exhibit a robust aerobic glycolytic capacity, and myeloid cells show a preference for the glycolytic metabolic pathway over lymphocytes (9). Upon infecting macrophages, bacteria initiate a metabolic reprogramming process that leads to a shift from oxidative phosphorylation to aerobic glycolysis. This metabolic remodeling is thought to be a critical defense mechanism employed by macrophages to combat infection (10). It provides the necessary energy and metabolic intermediates for macrophages to mount an effective immune response, including processes such as phagocytosis, cytokine production, and antigen presentation. Understanding the intricate relationship between glucose metabolism and macrophage function during bacterial infection is crucial for elucidating the underlying mechanisms of host defense and developing novel therapeutic strategies against infectious diseases.

Macrophages play a multifaceted role in the immune system, functioning in the phagocytosis and clearance of bacteria, as well as the modulation of immune metabolism. They achieve this by recognizing pathogen-associated molecular patterns and damage-associated molecular patterns through pathogen-recognition receptors (PRRs) (11). The PRR family encompasses several key members, including the Toll-like receptor, NOD-like receptor (NLR), C-type lectin receptor, and RIG-1-like receptor (12). Upon activation, PRRs initiate a cascade of events that further mediate the activation of signaling pathways integral to the innate immune response. These pathways include host transcriptional regulatory signaling, which controls the expression of genes involved in immune defense; inflammatory vesicle activation signaling, which is crucial for the release of inflammatory mediators; and mammalian target of rapamycin (mTOR) signaling, which regulates cell growth, metabolism, and autophagy. Through these activated signaling pathways, macrophages are able to regulate their resistance mechanisms against pathogens (13). The NLR, an important constituent of the PRR family, is predominantly expressed in the cytoplasm of macrophages (14). One of its key functions is to mediate the secretion of interleukin-1β (IL-1β) (15). Inflammasomes have been implicated in a wide range of autoimmune diseases, such as neurodegenerative disorders including multiple sclerosis, Alzheimer’s disease, and Parkinson’s disease, as well as metabolic disorders like atherosclerosis, type 2 diabetes mellitus, and obesity. In these pathological conditions, inflammasomes can act as either causative agents, directly contributing to disease initiation, or as promotive factors, exacerbating the disease progression (16).

In this study, our objective was to elucidate the role of NOD-like receptor 3 (NLRP3) in modulating macrophage glycolysis against *V. vulnificus* infection. We revealed that upon *V. vulnificu*s infection, NLRP3-deficient (NLRP3 knockout [KO]) macrophages exhibited reduced aerobic glycolysis compared to parental controls. Concurrently, there were significant decreases in glucose uptake, reactive oxygen species (ROS) production, and extracellular lactate levels. The current data indicate a possible involvement of NLRP3 signaling in modulating macrophage metabolic reprogramming in response to *V. vulnificus* infection. These results may provide a foundation for further investigation into the relationship between inflammasome activation and metabolic adaptation during bacterial infection.

## RESULTS

### Transcriptomic analysis reveals the metabolic reprogramming feature of J774A.1 macrophages following *V. vulnificus* infection

**Fig 1:**
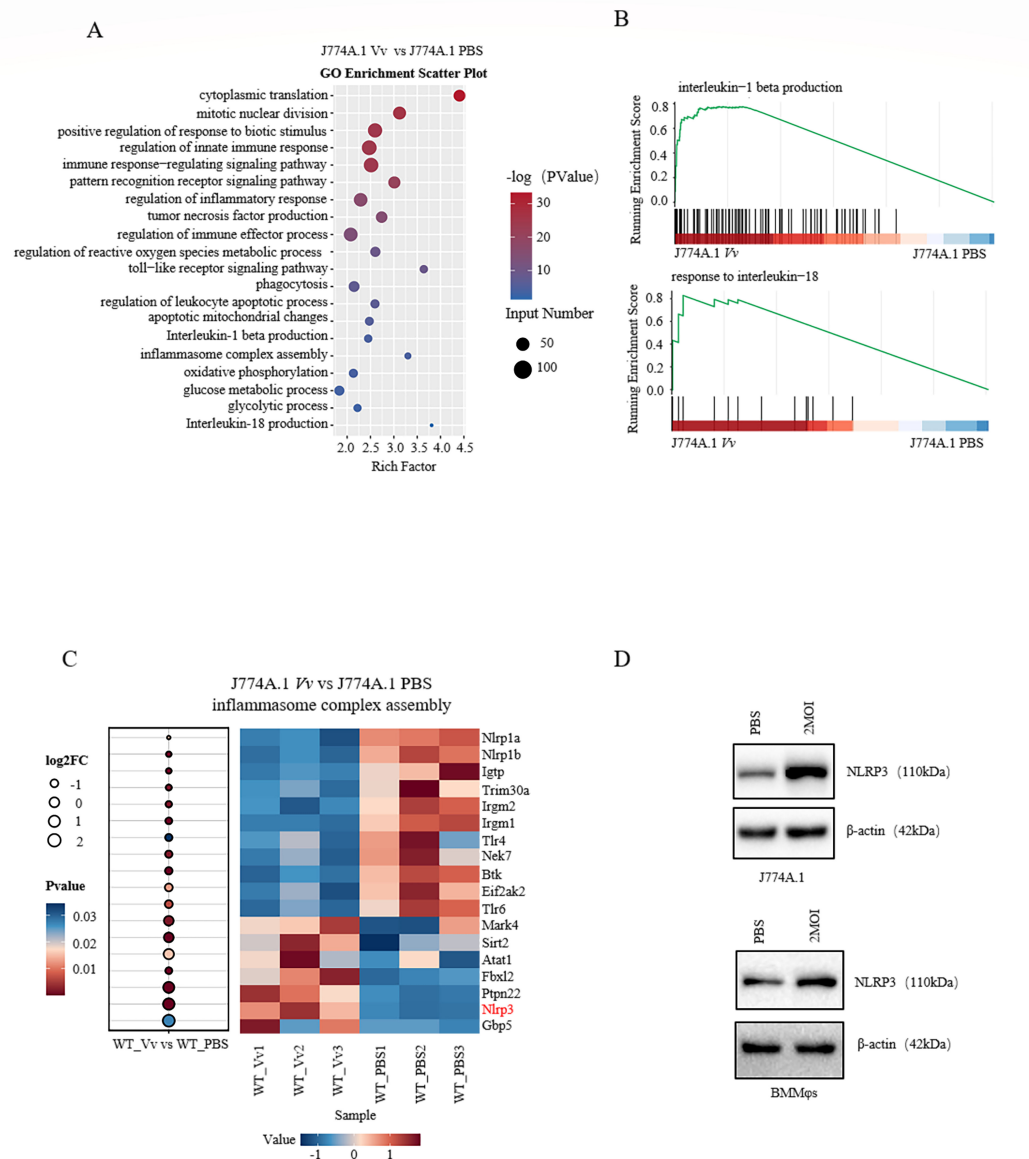
Transcriptomic profile of macrophages following *V. vulnificus* infection. (**A**) Enriched pathways by GO analysis of differentially expressed genes in *V. vulnificus* infected J774A.1. (**B**) Enrichment of IL-1β production and response to IL-18 in *V. vulnificus*-infected J774A.1 cells as revealed by GSEA. (**C**) Heatmap depicting the expression levels of genes involved in inflammasome complex assembly in RNA-Seq data. (**D**) Immunoblots showing the expression of NLRP3 protein in J774A.1 cells and BMMφs infected with *V. vulnificus* at 2 MOI.

The innate immune system serves as the host’s first line of defense against pathogenic microorganisms, playing a pivotal role in the clearance of pathogenic infections and the mitigation of endogenous threats. To decipher the mechanism underlying the innate immune response of macrophages to *V. vulnificus* infection, J774A.1 macrophages were infected with *V. vulnificus* at a multiplicity of infection (MOI) of 2 or treated with phosphate-buffered saline (PBS). Subsequently, the cells were harvested after 4 h for high-throughput RNA sequencing (RNA-Seq). Gene Ontology (GO) enrichment analysis of the differentially expressed genes revealed that pathways associated with inflammatory cytokines, such as those involved in interleukin-1β production and interleukin-18 production, were significantly enriched (Fig. 1A). It is well-established that macrophages, upon stimulation with lipopolysaccharide (LPS), do not exhibit rapid proliferation but instead produce a diverse array of inflammatory cytokines (17). Thus, gene set enrichment analysis (GSEA) was conducted, with a specific focus on the IL-1β and IL-18 gene sets. The results indicated that the expression of these gene sets was upregulated (using the NCBI Gene database; *P* < 0.05, normalized enrichment score >0; Fig. 1B). NLRs are known to sense pathogen-derived products or endogenous danger signals, leading to the activation of caspase-1. Caspase-1 cleaves gasdermin D (GSDMD), thereby inducing pyroptosis and facilitating the maturation of IL-1β and IL-18. The pores formed by GSDMD then release IL-1β and IL-18 into the extracellular space, where they exert their pro-inflammatory physiological functions (18). By comparing the expression levels of differential genes within the inflammasome complex assembly pathway between the control group and the *V. vulnificus*-infected J774A.1 macrophages, it was found that *Nlrp3* was highly expressed and showed significant differences (Fig. 1C). Subsequently, the expression of NLRP3 protein in macrophages following *V. vulnificus* infection was examined *in vitro*. The results demonstrated that the expression of NLRP3 protein was upregulated in both J774A.1 cells and murine bone marrow-derived macrophages after *V. vulnificus* infection *in vitro* (Fig. 1D). Collectively, these data indicate that NLRP3 might contribute to macrophage-mediated defense against *V. vulnificus* infection.

### Transcriptome analysis reveals NLRP3’s involvement in cell metabolism

**Fig 2:**
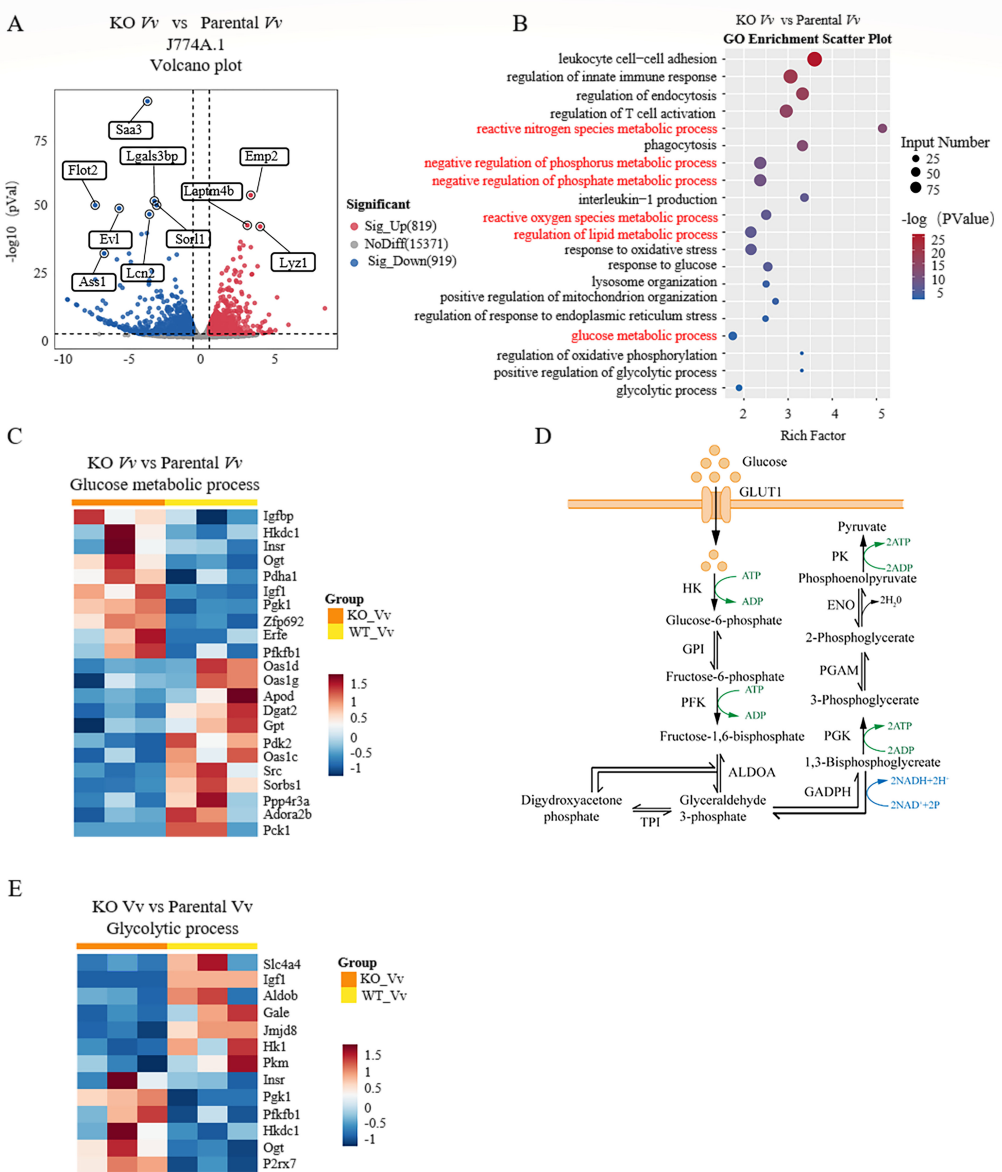
Involvement of NLRP3 in cellular metabolic processes as revealed by RNA-Seq analysis. (**A**) Volcano plot depicting differentially expressed genes between NLRP3 KO and parental J774A.1 cells post-*V. vulnificus* infection. (**B**) Enriched pathways identified by GO analysis of differentially expressed genes in *V. vulnificus*-infected J774A.1 cells. (**C**) Enrichment of glucose metabolic process and glycolytic process in *V. vulnificus*-infected parental J774A.1 cells as shown by GSEA. (**D**) Schematic of glycolysis pathway. (**E**) Heatmap illustrating the expression levels of genes associated with the glycolytic process in RNA-Seq.

To further explore the essential role of NLRP3 in *V. vulnificus*-infected macrophages, we employed the CRISPR-Cas9 system to generate an NLRP3 KO J774A.1 cell line. This cell line has been previously utilized in our studies (19). Subsequently, RNA-Seq analysis was carried out to obtain the transcriptomic profiles following *V. vulnificus* infection in the context of NLRP3 deficiency. The comparison between the NLRP3 KO and parental J774A.1 cell groups identified 1,738 differentially expressed genes. These genes exhibited a change of more than 1.5-fold and had a *P*-value <0.05, with 819 genes being upregulated and 919 genes being downregulated. Genes associated with immune defense and the inflammatory response (20, 21), such as *Saa1*, *Flot2*, and *Lcn2*, were significantly downregulated, as depicted in the volcano plot (Fig. 2A). GO enrichment analysis indicated that multiple metabolic pathways were significantly enriched (Fig. 2B). Based on these findings, we hypothesized that NLRP3 might enhance macrophage immune function by regulating metabolic reprogramming, thereby modulating the inflammatory response. A recent study has demonstrated that glycolysis in macrophages can trigger the activation of the NLR family and NLRP3 inflammasome (22). We compared the differential genes involved in the glucose metabolic process and the glycolytic process in NLRP3 KO and parental J774A.1 cells following 4 h of *V. vulnificus* infection. The expression of genes related to glucose utilization, such as *Pck*, *Scr*, and *Slc4a4*, was downregulated (Fig. 2C) (23–25). In glycolysis, hexokinase (HK), which encodes the first rate-limiting enzyme of glycolysis, exhibited strong inhibition in the *V. vulnificus*-infected NLRP3 KO cell model. Other glycolysis-related genes, such as ALDOB and PKM, were also coordinately downregulated (Fig. 2D and E). Our transcriptomic analysis of NLRP3 KO J774A.1 cells revealed that both glucose utilization and glycolysis levels were reduced after *V. vulnificus* infection. This suggests that NLRP3 may play a role in regulating the metabolic processes related to glucose utilization and glycolysis in macrophages during *V. vulnificus* infection.

### The effect of NLRP3 on the macrophage landscape of murine liver via single-cell sequencing analysis

**Fig 3:**
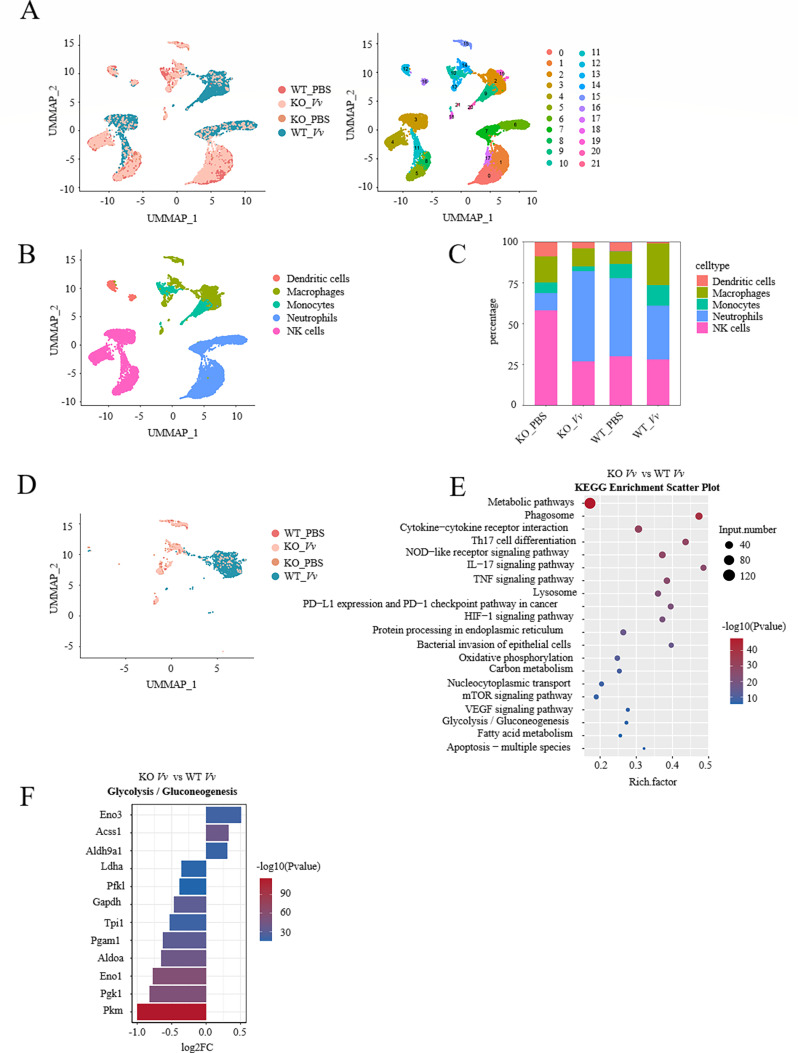
NLRP3 upregulates liver macrophage glycolysis in *V. vulnificus*-infected iron-overload mice. (**A**) UMAP representation of cellular clusters and experimental groups. (**B**) UMAP plot depicting annotated cell types. (**C**) The percentage of cell types in the indicated groups. (**D**) UMAP plot focused on macrophages across four experimental groups. (**E**) KEGG enrichment pathways scatter plot of differentially expressed genes in macrophages. (**F**) Bar chart illustrating fold change and *P*-values of glycolysis/gluconeogenesis-related differential expressed genes (DEGs) between NLRP3 KO and WT mice after *V. vulnificus* infection.

In consideration of the transcriptome analysis revealing a decreased glycolysis level in NLRP3 KO J774A.1 cells subsequent to *V. vulnificus* infection, we utilized the single-cell data of the livers of mice obtained from the previous stage of our studies (https://nmdc.cn/resource/genomics/sra/detail/NMDC40076921). These data, which were collected after *V. vulnificus* infection in an iron-overload mouse model, were employed to analyze the alterations in innate immune cell subsets within the murine liver. The resulting Uniform Manifold Approximation and Projection (UMAP) plot unveiled 21 primary cell clusters and their distribution across four groups (Fig. 3A). Each cluster was annotated based on the top 10 differential marker genes and subsequently classified into five cell types, namely macrophages, neutrophils, natural killer (NK) cells, dendritic cells, and monocytes (Fig. 3B). It is widely recognized that macrophages are the most abundant cell type within the tumor microenvironment (26), and these tumor-associated macrophages have been demonstrated to be the primary consumers of glucose in cancer (27). Following *V. vulnificus* infection, the proportion of macrophages in wild-type (WT) mice exhibited a significant increase, whereas in NLRP3 KO mice, the proportion of macrophages decreased (Fig. 3C and D). When comparing the differential genes between NLRP3 KO and WT murine liver macrophages after *V. vulnificus* infection, it was observed that metabolic pathways could be significantly enriched through Kyoto Encyclopedia of Genes and Genomes (KEGG) analysis (Fig. 3E). We opted to compare the differentially expressed genes within the glycolysis/gluconeogenesis pathways. Notably, among these genes, the key enzymes involved in the glycolytic reaction, such as pyruvate kinase (*Pkm*), phosphoglycerate kinase 1 (*Pgk1*), and enolase 1 (*Eno1*), exhibited downregulated expression in the liver macrophages of NLRP3 KO mice infected with *V. vulnificus* (Fig. 2D and 3F). The decrease in the expression of glycolytic-related genes in murine liver macrophages following NLRP3 KO was consistent with the results obtained from NLRP3 KO J774A.1 cells.

### NLRP3 regulates the glycolysis process in *V. vulnificus*-infected macrophages

**Fig 4:**
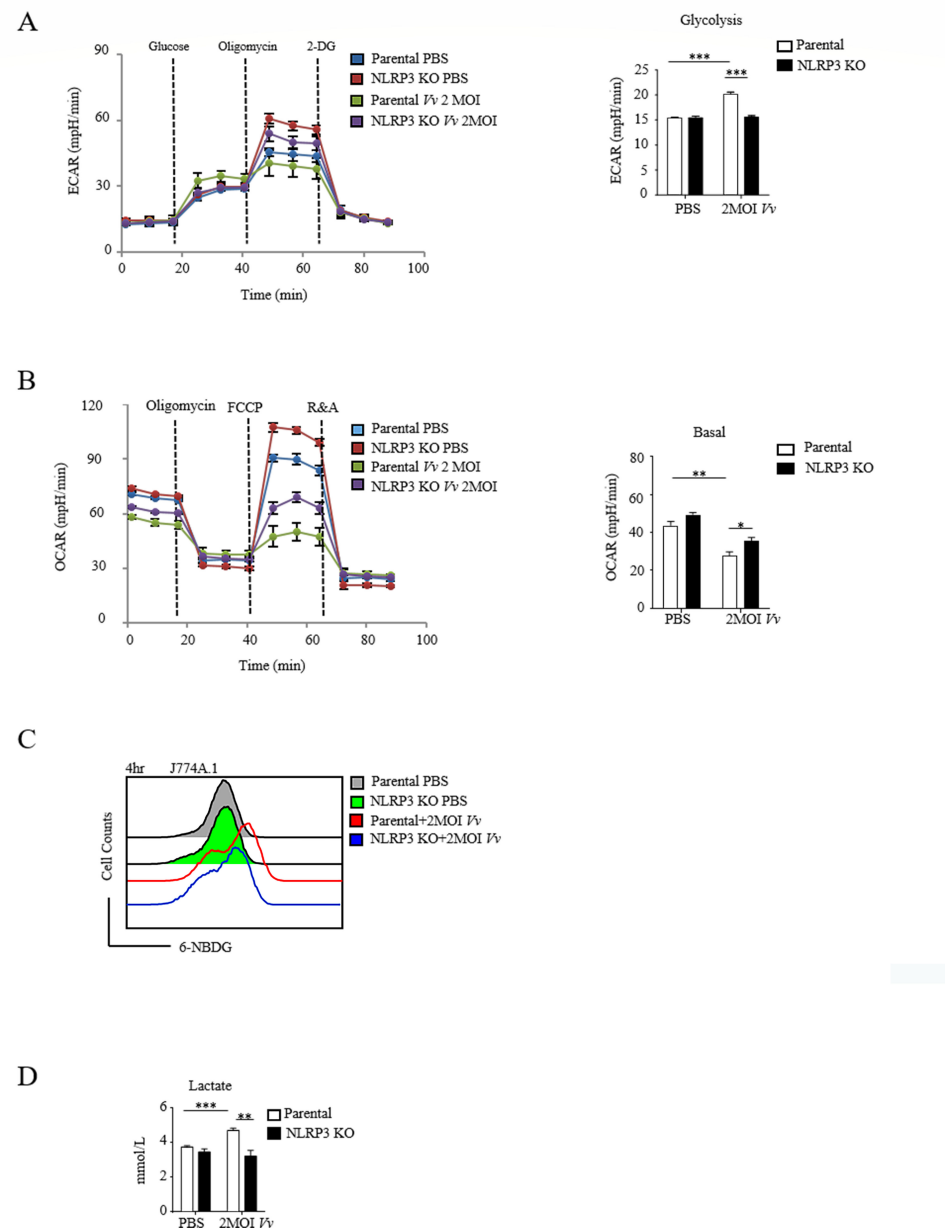
NLRP3 upregulates glycolysis in macrophages during *V. vulnificus* infection. (**A and B**) Seahorse analysis of metabolic parameters in parental and NLRP3 KO J774A.1 cells. Panel (**A**) shows ECAR and glycolytic capacity, and panel (**B**) shows OCR and mitochondrial respiratory capacity in parental and NLRP3 KO J774A.1 cells under basal conditions or following 4 h of infection with *V. vulnificus* at 2 MOI. (**C**) Flow cytometry analysis of glucose uptake in J774A.1 cells and splenic macrophages treated with 100 mM 6-[N-(7-nitrobenz-2-oxa-1,3-diazol-4-yl) amino]-6-deoxyglucose (6-NBDG) for the indicated times under basal conditions or after 4 h of 2 MOI *V. vulnificus* infection. (**D**) Lactate concentration in the supernatants of J774A.1 cells infected with *V. vulnificus* for 4 h. Data shown are representative of at least three experiments. *, *P* < 0.05; **, *P* < 0.01; ***, *P* < 0.001, determined by Student’s *t*-test.

Inflammation activation is intricately associated with metabolic reprogramming in macrophages (28). Specifically, activated macrophages undergo a metabolic reprogramming process wherein they shift from ATP production via OXPHOS to ATP production via glycolysis upon stimulation by LPS (29). Through bioinformatics enrichment analysis of the aforementioned sequencing results, we discovered that the glucose metabolic pathways in NLRP3 knockout J774A.1 cells following *V. vulnificus* infection were significantly inhibited. We analyzed both NLRP3 KO and parental J774A.1 macrophages for changes in the extracellular acidification rate (ECAR) and the mitochondrial oxygen consumption rate (OCR), which serve as indicators of glycolysis and OXPHOS, respectively. We observed that in parental J774A.1 cells, the ECAR increased upon infection, indicating enhanced glycolysis, accompanied by a reduction in the cellular OCR, which demonstrated the restoration of basal respiration. These results are consistent with the metabolic reprogramming characteristics of macrophages transitioning from glycolysis to oxidative phosphorylation following *V. vulnificus* infection. Moreover, we found that the infection of NLRP3 KO macrophages with *V. vulnificus* led to glycolytic levels comparable to those observed in macrophages treated with PBS. Additionally, the basal OCR of NLRP3 knockout macrophages still decreased after *V. vulnificus* infection, but the extent of this decrease was less than that of parental macrophages. This suggests that the metabolic effects of NLRP3 on OCR might be mediated by other mechanisms involved in OXPHOS regulation (Fig. 4A and B). These data imply that macrophages may enhance aerobic glycolysis through NLRP3.

Based on the increase in glycolytic flux indicated by ECAR, we examined the glucose uptake capacity of J774A.1 cells 4 h after treatment with *V. vulnificus in vitro* to analyze the effect of NLRP3. The flow cytometry histogram for 6-[N-(7-nitrobenz-2-oxa-1,3-diazol-4-yl) amino]-6-deoxyglucose (6-NBDG) shows a single population of cells whose fluorescence rapidly increases upon *V. vulnificus* infection. Compared with parental cells, the glucose uptake of NLRP3 KO cells infected by *V. vulnificus* was decreased (Fig. 4C), indicating that NLRP3 can promote the glucose uptake of macrophages infected by *V. vulnificus*. We further quantified the level of lactate, which is a product of glycolysis and anaerobic respiration, in the cell supernatants (Fig. 4D). In summary, these findings are consistent with a model where NLRP3 promotes aerobic glycolysis, possibly elevating glucose consumption and lactate production, which may influence macrophage metabolic adaptation and function in response to *V. vulnificus* infection.

### NLRP3-regulated glycolytic metabolism through phosphofructokinase-1 (PFK1) after *V. vulnificus* infection

**Fig 5:**
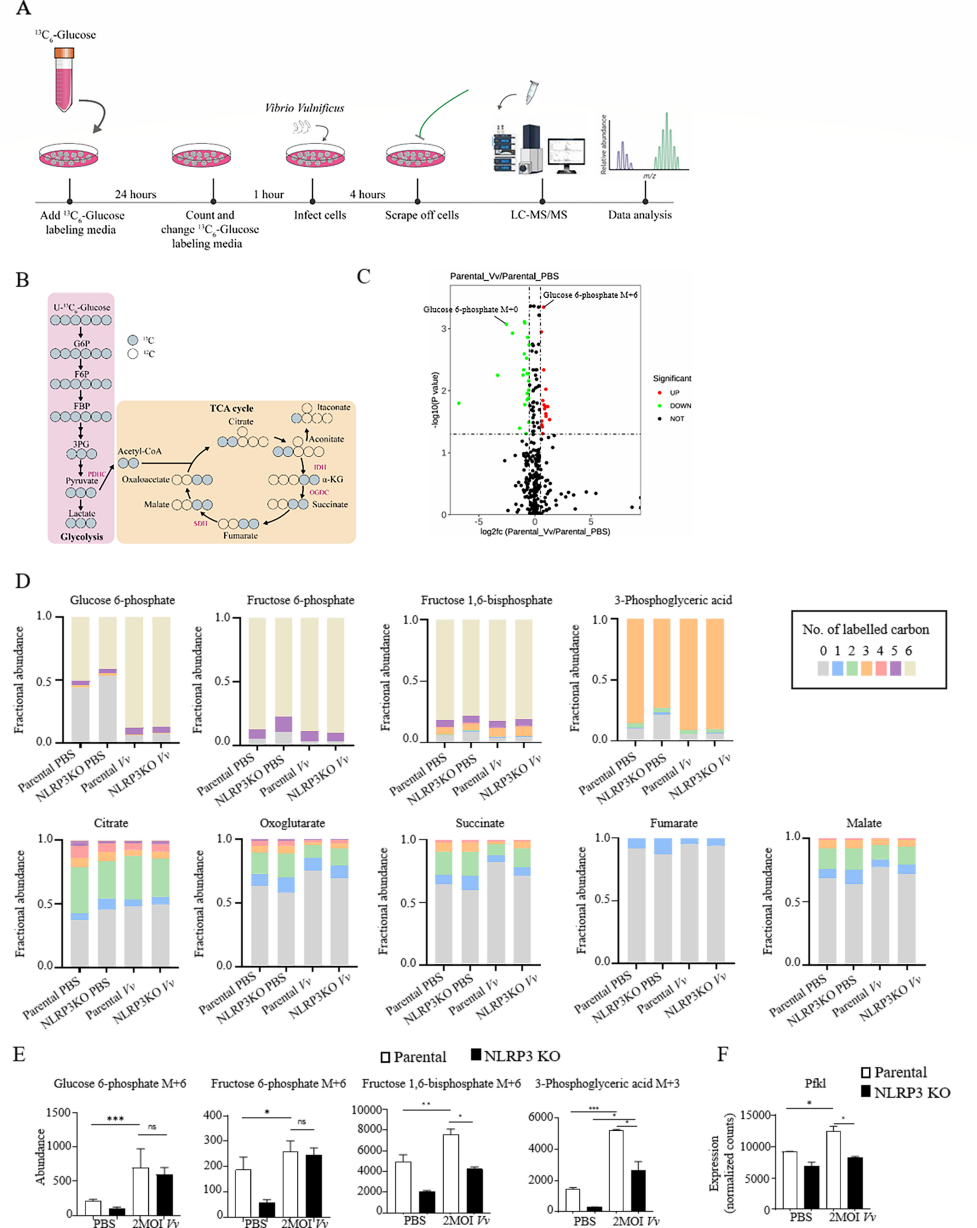
NLRP3-regulated glycolytic metabolism through PFK1 after *V. vulnificus* infection. (**A**) Schematic of workflow for the ^13^C_6_-glucose tracing experiment. (**B**) Tracer scheme illustrating the flux of U-^13^C_6_-glucose to different glycolytic branches. Blue circle: ^13^C; hollow circle: ^12^C. (**C**) Volcano plot showing metabolite changes. Metabolites in glucose metabolism showing significant changes are identified by name, and significantly changed metabolites are shown as red and green dots. Other changed metabolites are shown as black dots. (**D**) Labeling pattern of intracellular metabolites in parental and NLRP3 KO J774A.1 cells following stimulation with PBS and *V. vulnificus*. Bar graph represents the mean value. (**E**) ^13^C abundance of different glycolytic metabolites after U-^13^C_6_-glucose labeling in J774A.1 cells, which were treated with PBS or *V. vulnificus* infection for 4 h to labeling. (**F**) The mRNA expression of *Pfkl* in parental and NLRP3 KO J774A.1 cells through RNA-Seq after 4 h of 2 MOI *V. vulnificus* infection. *, *P* < 0.05; **, *P* < 0.01; ***, *P* < 0.001 was determined by Student's *t*-test.

Based on the transcriptomics and experimental data, we next explored the functional impact of NLRP3 inhibition on the cell metabolism of macrophages infected with *V. vulnificus* by conducting liquid chromatography-tandem mass spectrometry (LC-MS/MS) for steady-state levels of 58 metabolites (Fig. 5A). Among the diverse dynamic behaviors in different metabolites, we were particularly interested in whether the transient increase in glycolytic intermediates regulates the inflammatory status of macrophages. We subsequently used uniformly labeled ¹³C₆-glucose (U-¹³C₆-glucose) to trace glucose flux into different metabolic pathways in response to parental and NLRP3 KO J774A.1 cells after *V. vulnificus* infection (Fig. 5B). After 4 h of *V. vulnificus* infection, the fraction of M+6 labeled glucose 6-phosphate was dramatically increased, and unlabeled glucose 6-phosphate was dramatically reduced compared to unstimulated macrophages (Fig. 5C). The labeling of glycolysis metabolites downstream of glucose 6-phosphate, such as fructose 6-phosphate, fructose 1,6-bisphosphate, and 3-phosphoglyceric acid, also reflected this accumulation of labeling, whereas total labeling of TCA cycle metabolites, such as citrate, oxoglutarate, succinate, fumarate, and malate, remained lower after infection (Fig. 5D). The increased glycolysis labeling and decreased TCA cycle labeling suggested reduced relative flux into the TCA cycle from glycolysis. Following a 4-hour exposure to infection, NLRP3 knockout cells displayed a significantly greater fraction of labeled TCA cycle metabolites, including citrate, relative to parental cells. However, the relative abundance of glycolytic metabolites in NLRP3 KO cells following *V. vulnificus* infection showed no significant difference compared to parental macrophages (Fig. 5D). This observation may be attributed to the pronounced upregulation of glycolytic intermediates post-infection, which could diminish proportional differences between the two groups. So we quantitatively analyzed absolute abundance changes of key metabolites in glycolysis. Following 4 h of infection, metabolic analysis of NLRP3 KO J774A.1 cells revealed a pronounced reduction in the utilization of uniformly labeled ¹³C₆-glucose for glycolytic metabolite production, such as fructose 1,6-bisphosphate M+6 and 3-phosphoglyceric acid M+3, whereas glucose 6-phosphate M+6 and fructose 6-phosphate M+6 showed no difference between parental and NLRP3 KO cells after infection (Fig. 5E). PFK1 is the key rate-limiting enzyme that converts fructose 6-phosphate into fructose 1,6-bisphosphate (30). It was reported that PFKL, one isomer of PFK1, was crucial in regulating the changes of macrophage glycolysis after LPS treatment (31). So we found that the transcription of PFKL was decreased in *V. vulnificus*-infected NLRP3 KO J774A.1 cells compared to parental J774A.1 cells after *V. vulnificus* infection (Fig. 5F). The results suggested that NLRP3 might regulate the glycolytic process of macrophages through PFKL after *V. vulnificus* infection.

### NLRP3 promotes ROS production in macrophages following *V. vulnificus* infection

**Fig 6:**
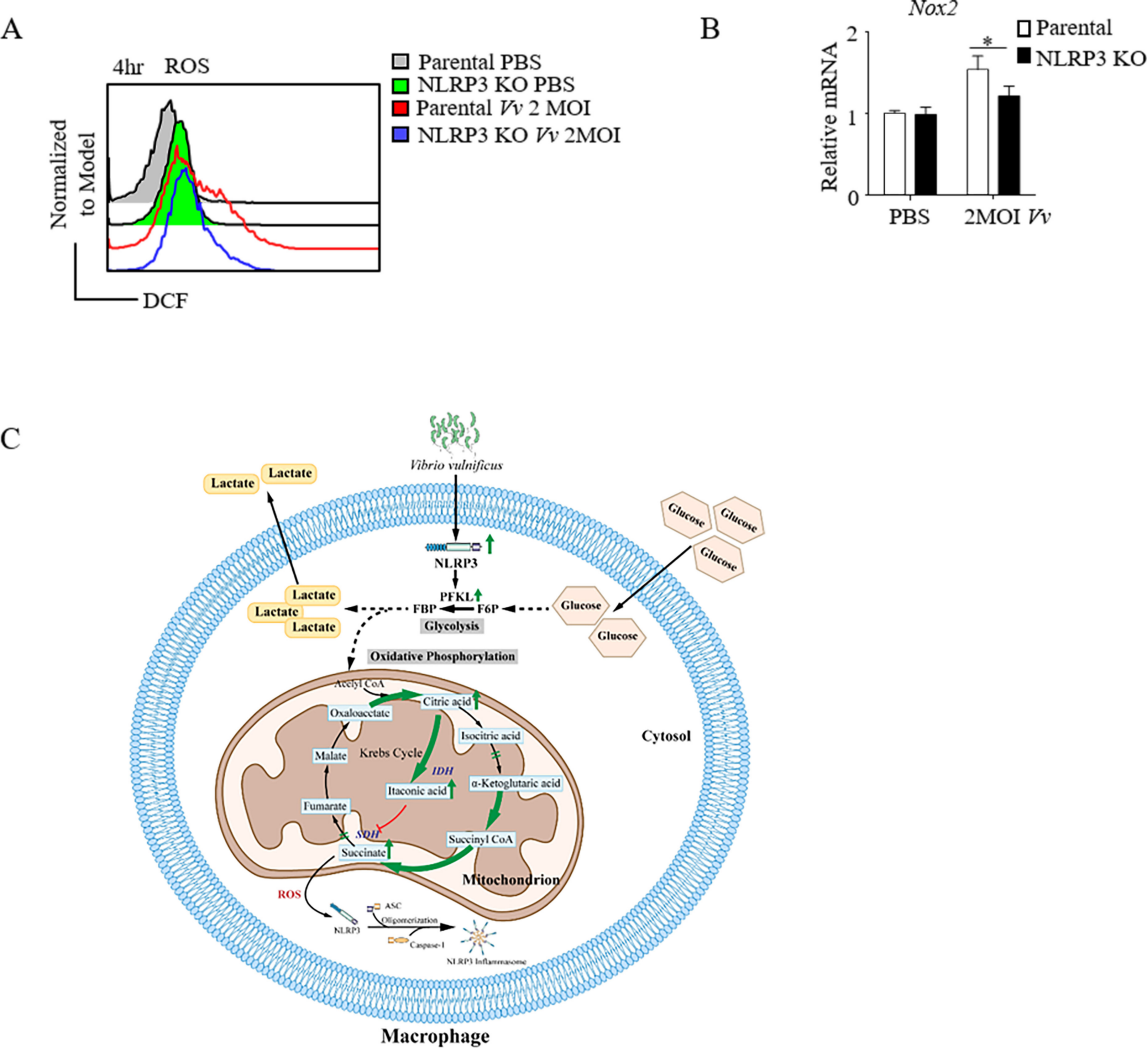
NLRP3 upregulates ROS production in macrophages during *V. vulnificus* infection. (**A**) ROS production was measured in NLRP3 KO and parental J774A.1 cells under 2 MOI *V. vulnificus* infection for 4 h by flow cytometry. (**B**) RT-qPCR analysis of *Nox2* transcription in NLRP3 KO and parental J774A.1 cells. (**C**) Graphic abstract: NLRP3 regulates the activation of glycolysis via glucose uptake, lactate release, and ROS production in *V. vulnificus*-infected macrophages. *, *P* < 0.05 was determined by Student’s *t*-test.

ROS has long been recognized as being fundamental for macrophages to eliminate invasive microorganisms through the oxidative burst mediated by nicotinamide adenine dinucleotide phosphate (NADPH) oxidase (32). However, more recent investigations have demonstrated that mitochondrial ROS play essential roles in several innate immune functions by virtue of subtle changes in the intracellular redox state (33). The intracellular levels of ROS were evaluated 4 h post-infection using the 2′,7′-Dichlorodihydrofluorescein diacetate(DCFH-DA) probe. *V. vulnificus* infection was found to exert significant impacts on the intracellular levels of ROS. In comparison with parental cells, the intracellular ROS production of NLRP3 KO cells infected by *V. vulnificus* was decreased(Fig. 6A). NADPH oxidases have been identified as one of the key sources of ROS in immune cells. In particular, the NOX2 NADPH oxidase has been attributed multiple roles, serving as a source of antimicrobial ROS (34). This is evidenced by the higher expression of *Nox2* upon *V. vulnificus infection* (Fig. 6B). Upon *V. vulnificus* infection, NLRP3 KO led to a reduction in both ROS production and *Nox2* expression levels compared with parental cells. Our data imply that NLRP3 may contribute to ROS generation in macrophages during *V. vulnificus* infection, potentially playing a role in the host defense against this pathogen. In summary, we show that *V. vulnificus* infection can activate macrophages, resulting in metabolic reprogramming from oxidative phosphorylation to aerobic glycolysis. Meanwhile, the expression of NLRP3 in macrophages increased significantly after infection. We found that the activation of glycolysis is closely related to NLRP3. In particular, NLRP3 can promote glucose uptake, upregulate aerobic glycolytic pathway, promote lactic acid release, and produce ROS, thus affecting immune response in macrophages against *V. vulnificus* (Fig. 6C).

## DISCUSSION

An increasing number of studies have established the intricate relationship between intracellular metabolism and inflammation. The activation of immune cells is invariably accompanied by alterations in intracellular metabolism, which in turn affects the functions of these immune cells. Once activated, immune cells undergo extensive metabolic reprogramming to meet the substantial increase in energy requirements and to support various immune-related functions, such as the robust production of cytokines, rapid proliferation, and migration activities (17). Notably, the escalating demand for glucose and the upregulation of the aerobic glycolysis rate are pivotal components of the intracellular metabolic transformation triggered by pro-inflammatory signals. This transformation redirects glucose metabolism toward lactic acid production instead of the mitochondrial TCA cycle. In the context of immune metabolism, the coupling of the mitochondrial TCA cycle and OXPHOS represents an efficient energy-generating process. However, glycolysis can be upregulated more rapidly to meet the energy demands of activated pro-inflammatory immune cells (35).

Our analysis of RNA-Seq data from J774A.1 macrophages revealed distinct differences in cytokine production, ROS generation, oxidative phosphorylation, and the glycolysis process following bacterial infection. Significantly, the liver serves as the central metabolic hub of the body, coordinating complex communication with various tissues through the secretion of hormones and metabolites. It is also uniquely adapted to maximize interactions between immune cells and pathogens within the bloodstream (36). Single-cell RNA-seq (scRNA-seq) data from the murine livers of NLRP3 KO and WT mice disclosed significant disparities in the composition of innate immune cells. In WT mice, the proportion of macrophages in the liver increased substantially after *V. vulnificus* infection. When specifically examining this macrophage cluster, compared with infected WT mice, NLRP3 KO mouse liver macrophages exhibited marked differences in metabolic pathways post-infection, particularly a downregulation of key enzymes in the glycolytic pathway. This finding is consistent with the transcriptome results obtained from cell lines. Our experiments indicate that *V. vulnificus* infection enhances macrophage glycolysis. Macrophages absorb a large quantity of glucose to efficiently generate energy and simultaneously secrete lactic acid into the extracellular space, as evidenced by an increased extracellular acidification rate and reduced oxygen consumption detected via Seahorse analysis. This metabolic profile is consistent with that of M1 macrophages, as observed by Hard et al. (37).

It is well-established that the enhancement of glycolysis is achieved through the upregulation of glycolytic enzymes, such as hexokinases 1 and 2 (HK1, HK2), glyceraldehyde-3-phosphate dehydrogenase, and pyruvate kinase isoenzyme M2 (PKM2), as well as an increase in the surface expression of the glucose transporter GLUT1 (38–41). Additionally, glycolysis supplies substrates for the pentose phosphate pathway (PPP) and synthesizes precursors for nucleotide, amino acid, and fatty acid synthesis, thereby supporting anabolic growth and cytokine secretion (17, 42). NADPH generated in the PPP is utilized by NADPH oxidase to rapidly produce ROS and for glutathione biosynthesis, which helps counteract oxidative stress (43). Moreover, due to the interruption of the TCA cycle, which leads to the accumulation of mitochondrial metabolites, macrophages modulate their transcriptional processes to enhance the expression of glycolytic enzymes and initiate inflammatory responses (44). Moreover, due to the interruption of the TCA cycle, which leads to the accumulation of mitochondrial metabolites, macrophages modulate their transcriptional processes to enhance the expression of glycolytic enzymes and initiate inflammatory responses (45). The basal activation of the cryopyrin-associated periodic syndrome-related NLRP3 inflammasome significantly modifies cellular metabolites, including lipids, lipid-like molecules, amino acids, and their intermediates (46). The GO enrichment analysis of our differential genes further corroborates these findings, as it shows that related biological processes and gene upregulation support this metabolic reprogramming. In this study, we discovered that NLRP3 deficiency in macrophages affects the expression of key glycolytic enzymes, such as Pkm and HK1, following infection. Previous studies have shown that PKM2-mediated glycolysis promotes inflammasome activation by modulating eukaryotic translation initiation factor 2 alpha kinase 2 (EIF2AK2) phosphorylation in macrophages. Inhibition of PKM2 or EIF2AK2 attenuates NLRP3 inflammasome activation, thereby suppressing the release of IL-1β, IL-18, and high mobility group box 1 (HMGB1) by macrophages. Moon et al. demonstrated that hexokinase 1-dependent glycolysis, under the regulation of mammalian target of rapamycin complex 1 (mTORC1), represents a critical metabolic pathway for NLRP3 inflammasome activation. Glucose deprivation, glycolysis inhibition (e.g., treatment with 2-deoxyglucose), or silencing of Hk1 inhibits caspase-1 activation and IL-1β secretion (39). However, the question of whether NLRP3 inflammasome activation can regulate cellular glycolysis remains incompletely understood.

Our previous studies have revealed that the bactericidal activity of macrophages in internalizing *V. vulnificus* is contingent upon the interaction between the mTOR and the NLRP3. The absence of NLRP3 impedes mTORC1 signal transduction, disrupts *V. vulnificus*-induced phagosome acidification and phagolysosome formation, ultimately resulting in a decline in intracellular bacterial clearance (19). Recent investigations have highlighted the critical role of mTOR activation in microglial cells. mTOR activation enables microglial cells to efficiently utilize glucose for energy production and respond to inflammatory signals. Inhibition of mTOR not only curbs the elevated glycolysis in microglial cells but also facilitates their transition from the M1 to the M2 phenotype, thereby reducing the production of inflammatory substances (47, 48). Based on these findings, we postulate that NLRP3 may modulate glycolysis through the Raptor/mTORC1 complex. When compared with parental macrophages, NLRP3 KO macrophages exhibit lower glucose uptake, reduced lactate release, and a decreased extracellular acidification rate. Moreover, metabolic profiling using U-¹³C₆-glucose revealed that the metabolic reaction from fructose 6-phosphate to fructose 1,6-bisphosphate was affected in *V. vulnificus*-infected NLRP3 KO J774A.1 cells, indicating that PFK1, the key rate-limiting enzyme that converts fructose 6-phosphate into fructose 1,6-bisphosphate, was probably regulated by NLRP3. In mammals, PFK1 is composed of three different isoforms as homodimers or heterodimers, each encoded by a distinct gene locus: PFKP (platelets), PFKL (liver), and PFKM (muscle) (49). PFKL is the only one that has been reported to undergo Ser775 phosphorylation after being activated by pattern recognition receptors in macrophages. This phosphorylation enhances glycolytic activity and promotes the production of pro-inflammatory factors such as IL-1β (50). The results showed that the transcriptional level of PFKL was decreased significantly in *V. vulnificus*-infected NLRP3 KO macrophages. These observations may imply that NLRP3 regulates the metabolic reprogramming of macrophages toward glycolysis via PFKL. These results indicated that NLRP3 deficiency might alter metabolic adaptation, with glucose potentially being utilized through alternative pathways to support macrophage function during infection.

It has been established that activated macrophages undergo Warburg-like metabolic reprogramming. When the tricarboxylic acid cycle is disrupted, the intermediate succinate accumulates. This increased succinate is then oxidized by succinate dehydrogenase, generating ROS. Subsequently, ROS further induces the activation and assembly of the NLRP3 inflammasome (51). The NADPH oxidase NOX2 complex, a transmembrane oxidase expressed in macrophages, is responsible for the oxidative burst required to eliminate ingested pathogens and is thus regarded as the primary source of ROS in inflammatory tissues (34). After NLRP3 knockout, both the production of ROS by macrophages and the expression of *Nox2* are reduced following *V. vulnificus* infection.

In summary, our findings demonstrate that NLRP3 plays a hitherto underappreciated role in the immune function of macrophages during *V. vulnificus* infection. Consequently, targeting NLRP3 represents a potential therapeutic approach for treating *V. vulnificus i*nfections. The expression of NLRP3 is upregulated in macrophages infected with *V. vulnificus*, and the absence of NLRP3 significantly downregulates the expression of inflammation-related genes. Our results indicate that NLRP3 may influence the immune response by regulating macrophage glycolysis. However, further research is warranted to comprehensively elucidate the impact of NLRP3 on macrophage glycolysis during *V. vulnificus* infection.

## MATERIALS AND METHODS

### Bacterial strains and cell culture

J774A.1 cells were purchased from the Cell Bank of Type Culture Collection of Chinese Academy of Sciences in Shanghai. NLRP3-deficient J774A.1 cell line was generated as previously described (19). All the cells were cultured in RPMI 1640 containing 10% heat-inactivated fetal bovine serum (Tianhang Bio) and penicillin-streptomycin (50 IU/mL and 50 mg/mL, Beyotime). The China General Microbiological Culture Collection Center provided the *V. vulnificus* ATCC 27562 strain. We grew *V. vulnificus* at 37°C in brain heart infusion (BHI) broth or on the BHI rabbit blood agar plate.

### Mice

Eight- to 10-week-old female C57BL/6J mice were purchased from Zhejiang Charles River Laboratory Animal Technology Company.

### J774A.1 cells infected with *V. vulnificus in vitro*

J774A.1 cells were plated in 35 mm dishes, and then *V. vulnificus* was added to the cells at the indicated MOI. The supernatant was collected at the indicated time for cytokine quantification. The infected cells were used for flow cytometry analysis, quantitative reverse transcription PCR analysis, and Western blot analysis.

### RNA sequencing

J774A.1 cells with 4 h *V*. *vulnificus* infection or PBS treatment were used for RNA sequencing. Briefly, the cells were lysed in TRIzol and subjected to Annoroad Co. for RNA extraction, sequencing, and transcriptome profile analysis. The significantly differentially expressed genes were identified when we compared the normalized reads count between *V. vulnificus* and PBS groups with *P* < 0.05 and |Log2FoldChange| > 0.585. The significance of the gene ontology term enrichment was estimated using Fisher’s exact test (*P*-value).

### Western blot analysis

Total protein extracts were produced using radioimmunoprecipitation assay buffer includingphenylmethylsulfonyl fluoride protease inhibitors (Beyotime Biotechnology, Shanghai, China) and a phosphatase inhibitor (Beyotime Biotechnology) from cultured cells. An Enhanced BCA Protein Assay Kit was used to measure protein (Epizyme Biotech). Equal quantities of protein were separated by SDS-PAGE and electro-transferred to polyvinylidene fluoride membranes (Merck Millipore, Billerica, MA, USA). The membrane was blocked at room temperature for 2 h with 5% nonfat milk. After blocking, the membranes were incubated with primary antibodies against NLRP3 and β-actin (1:1,000; Cell Signaling Technology) overnight at 4°C. Then, the secondary antibodies HRP Goat Anti-Rabbit IgG (H+L) (1:3,000; Beyotime Biotechnology) or HRP Goat Anti-Mouse IgG (H+L) (1:3,000; Beyotime Biotechnology) were used to incubate the membranes for 1 h at room temperature. The protein expression was detected using a chemiluminescent detection system (Bio-Rad) after adding the ECL Enhanced Plus Kits (Epizyme Biotech).

### Glucose uptake

After different interventions, 100 μM 6-NBDG was added to J774A.1 macrophages, which were cultured in a 5% CO_2_ cell incubator at 37°C for 30 min by RPMI 1640 without glucose, and then washed with PBS three times. Cells were collected and suspended in precooled PBS. The fluorescence signal values of 20,000 cells in each group were detected by flow cytometry and analyzed quantitatively.

### *V. vulnificu*s-infected mouse model

Each mouse was intraperitoneally (i.p.) injected with 200 µg/g of iron-dextrin (purchased from Thermo Fisher). Two hours post-injection of iron-dextrin, the mice were then i.p. injected with either 2 × 10⁵ colony-forming units of *V. vulnificus* suspended in 200 µL of PBS or with 200 µL of PBS alone. Seven hours after the second injection (infection), the mice were euthanized and bled to facilitate the collection of blood and tissue samples.

### Single-cell RNA library preparation and sequencing

The live/dead^-^, CD3^-^, CD19^-^, CD49^-^, and CD45^+^ cells in the murine liver were sorted by MoFlo Astrios cell sorter (Beckman) and processed through a 10× Genomics machine and then through a library preparation by LC Sciences, following the standard protocol of Chromium Single Cell 3′ Reagent Kit (v2 Chemistry) (10× Genomics, Cat#: CG00052, California, USA). These libraries were analyzed utilizing the NovaSeq 6000 for Illumina sequencing (LC-Bio Technology, Hangzhou, China).

### scRNA-Seq data processing

Sample demultiplexing, barcode processing, alignment to the mouse genome (specifically, the Ensembl mm10 genome, version 105), and the counting of raw gene expression were carried out using the CellRanger software (version 7.1.0; 10× Genomics, USA), as detailed in the official documentation available at (expression/software/overview/welcome). The raw single-cell expression matrices were subjected to in-depth analysis using the R software (version 4.1.2) in conjunction with the Seurat package (version 4.1.1). As a crucial initial step, rigorous quality control measures were implemented to eliminate low-quality cells and genes with suboptimal expression levels.

### Non-targeted metabolomics

#### Isotope labeling

U-^13^C_6_-glucose labeling J774A.1 cells were maintained in the indicated treatment for 24 h, at which point labeled media (glucose-free RPMI [Thermo Fisher Scientific] supplemented with 10% serum and 2 g/L U-^13^C_6_-glucose [Cambridge Isotopes]) was replaced. For glucose-flux analysis, cells were cultured in the labeled media for 4 h with PBS treatment or *V. vulnificus* infection.

#### LC-MS/MS

Non-targeted metabolic flux analysis was conducted at LipidALL Technologies as described previously (52–54). Polar metabolites were extracted using ice-cold methanol containing phenylhydrazine. Samples were incubated at 1,500 rpm for 30 min at 4°C. Following the incubation, samples were kept at −20 ℃ for 1 h for derivatization of alpha-keto acids (54). Then, samples were centrifuged for 15 min at 12,000 rpm and 4°C. The clean supernatant was transferred to a new tube and dried in a SpeedVac. Total protein content was determined from the dried pellet using the Pierce BCA Protein Assay Kit according to the manufacturer’s protocol. The dried extract was reconstituted in 5% acetonitrile in water prior to LC-MS analysis on an Agilent 1290 II UPLC coupled to Sciex 5600+ quadrupole-time of flight (TOF) MS. For reverse-phase liquid chromatography (RPLC), polar metabolites were separated on a Waters ACQUITY HSS-T3 column (3.0 × 100 mm, 1.8 µm), while a Waters ACQUITY BEH Amide column (2.1 × 100 mm, 1.7 µm) was utilized for hydrophilic interaction liquid chromatography (HILIC). MS parameters for detection were electrospray ionization source voltage negative ion mode −4.5 kV; vaporizer temperature, 500°C; drying gas (N_2_) pressure, 50 psi; nebulizer gas (N_2_) pressure, 50 psi; curtain gas (N_2_) pressure, 35 psi. The scan ranges were set at m/z 60–700 during RPLC and m/z 70–850 during HILIC analysis, respectively. Information-dependent acquisition mode was used for MS/MS analyses of the metabolites. Collision energy was set at (−) 35 ± 15 eV. Data acquisition and processing were performed using Analyst TF 1.7.1 Software (AB Sciex, Concord, ON, Canada). All detected ions were extracted using MarkerView 1.3 (AB Sciex, Concord, ON, Canada) into Excel in the format of a two-dimensional matrix, including mass-to-charge ratio (m/z), retention time, and peak areas. PeakView 2.2 (AB Sciex, Concord, ON, Canada) was applied to extract MS/MS data and perform comparisons with the metabolites database (AB Sciex, Concord, ON, Canada), human metabolome database, and standard references to annotate ion identities (52, 53). To correct for inter-sample variations in ionization efficiency, L-leucine-d10 was used as an internal standard for normalization of peak areas across samples.

### ROS detection

J774A.1 macrophage was suspended in 1 mL of DCFH-DA probe at a final concentration of 10 µmol/L and cultured in a 5% CO_2_ cell incubator at 37 ℃ for 20 min. During incubation, the cell flipped upside down every 5 minutes, in order to make the probe fully contact with the cells. Cells were collected and washed with PBS three times, then were subjected to flow cytometry analysis.

### Statistical analysis

Data were presented as mean ± SEM and analyzed for statistical differences using the Prism 8.0/GraphPad software. Statistical significance was analyzed using the Student’s *t*-test. *P*-values less than 0.05 were considered significant.

## Data Availability

The raw data supporting the conclusions of this article will be made available by the authors, without undue reservation, to any qualiﬁed researcher. The scRNASeq datasetsdata sets generated and/or analyzed during the current study are available in the National Microbiology Data Center (NMDC) with the accession number NMDC40076921. The RNASeq datasetsdata sets generated and/or analyzed during the current study are available in the NCBI Sequence Read Archive (SRA) data with the accession number PRJNA1166737. The non-targeted metabolomics data is deposited in National Microbiology Data Center (NMDC) with accession numbers NMDC10019953.

## SUPPLEMENTAL MATERIAL

Abbreviation spectrum.00230-25-s0001.docx Abbreviation for the gene names in metabolism.

ASM does not own the copyrights to Supplemental Material that may be linked to, or accessed through, an article. The authors have granted ASM a non-exclusive, world-wide license to publish the Supplemental Material files. Please contact the corresponding author directly for reuse.
