# Supplementary material for: NLRP3 is crucial for macrophage metabolic reprogramming during Vibrio vulnificus infection
Source: Microbiol Spectr. 2025 Nov 5;13(12):e00230-25. doi: 10.1128/spectrum.00230-25 (PMC12671130; doi:10.1128/spectrum.00230-25)
Supplement: Abbreviation — for the gene names in metabolism. [file spectrum.00230-25-s0001.docx]

**Source：**[**http://www.informatics.jax.org**](http://www.informatics.jax.org/)

**Fig1C**

**Nlrp1a: NLR family, pyrin domain containing 1A**

**Nlrp1b: NLR family, pyrin domain containing 1B**

**Igtp: Immunity Related GTPase**

**Trim30a:  tripartite motif-containing 30A**

**Irgm2：Immunity-Related GTPase Family M Member 2**

**Irgm1：Immunity-Related GTPase Family M Member 1**

**Tlr4：toll-like receptor 4**

**Nek7：NIMA-related expressed kinase 7**

**Btk：Bruton agammaglobulinemia tyrosine kinase**

**Eif2ak2： eukaryotic translation initiation factor 2-alpha kinase 2**

**Tlr6：toll-like receptor 6**

**Mark4：MAP/microtubule affinity regulating kinase 4**

**Sirt2：sirtuin 2**

**Atat1：alpha tubulin acetyltransferase 1**

**Fbxl2：F-box and leucine-rich repeat protein 2**

**Ptpn22：protein tyrosine phosphatase, non-receptor type 22**

**Nlrp3： NLR family, pyrin domain containing 3**

**Gbp5：guanylate binding protein 5**

**Fig2A**

**Saa3：serum amyloid A 3**

**Flot2：flotillin 2**

**Lgals3bp：Galectin-3 Binding Protein**

**Emp2：epithelial membrane protein 2**

**Laptm4b：lysosomal-associated protein transmembrane 4B**

**Evl：Ena-vasodilator stimulated phosphoprotein**

**Sorl1： sortilin related receptor 1**

**Ass1： argininosuccinate synthetase 1**

**Lcn2：lipocalin 2**

**Lyz1： lysozyme 1**

**Fig2C**

**Igfbp：insulin-like growth factor binding protein**

**Hkdc1：hexokinase domain containing 1**

**Insr：insulin receptor**

**Ogt：O-linked N-acetylglucosamine (GlcNAc) transferase**

**Pdha1： pyruvate dehydrogenase E1 alpha 1**

**Igf1：insulin-like growth factor 1**

**Pgk1：phosphoglycerate kinase 1**

**Zfp692：zinc finger protein 692**

**Erfe：erythroferrone**

**Pfkfb1：fructose-2,6-biphosphatase 1**

**Oas1d：2'-5' oligoadenylate synthetase 1D**

**Oas1g：2'-5' oligoadenylate synthetase 1G**

**Apod：apolipoprotein D**

**Dgat2：diacylglycerol O-acyltransferase 2**

**Gpt：glutamic pyruvic transaminase**

**Pdk2：Pyruvate Dehydrogenase Kinase 2**

**Oas1c：2'-5' oligoadenylate synthetase 1C**

**Src：Rous sarcoma oncogene**

**Sorbs1：sorbin and SH3 domain containing 1**

**Ppp4r3a：protein phosphatase 4 regulatory subunit 3A**

**Adora2b：adenosine A2b receptor**

**Pck1：phosphoenolpyruvate carboxykinase 1**

**Fig2D**

**Slc4a4：Solute Carrier Family 4 Member 4**

**Igf1： insulin-like growth factor 1**

**Aldob：aldolase B**

**Gale：UDP-galactose-4-epimerase**

**Jmjd8：jumonji domain containing 8**

**Hk1： hexokinase 1**

**Pkm：Pyruvate Kinase Muscle**

**Insr：insulin receptor**

**Pgk1：phosphoglycerate kinase 1**

**Pfkfb1：6-phosphofructo-2-kinase**

**Hkdc1：hexokinase domain containing 1**

**Ogt ：O-linked N-acetylglucosamine (GlcNAc) transferase**

**P2rx7：purinergic receptor P2X, ligand-gated ion channel, 7**

**Fig3F**

**Eno3：enolase 3, beta muscle**

**Acss1：acyl-CoA synthetase short-chain family member 1**

**Aldh9a1：aldehyde dehydrogenase 9, subfamily A1**

**Ldha：lactate dehydrogenase A**

**Pfkl：phosphofructokinase, liver, B-type**

**Gapdh：glyceraldehyde-3-phosphate dehydrogenase**

**Tpi1：triosephosphate isomerase 1**

**Pgam1：phosphoglycerate mutase 1**

**Aldoa：aldolase A, fructose-bisphosphate**

**Eno1：enolase 1, alpha non-neuron**

**Pgk1：phosphoglycerate kinase 1**

**Pkm：pyruvate kinase, muscle**
